# A clinicopathological prediction model for remission after transsphenoidal surgery for acromegaly: A retrospective single-center cohort study

**DOI:** 10.1097/MD.0000000000051358

**Published:** 2026-11-06

**Authors:** Rob A. Vergeer, Henriette E. Westerlaan, Annet Willems, Anke D. van den Beukel, Friederike E. Roelandt-Schumann, Henk J. Buikema, Jos M.A. Kuijlen, J. Marc C. van Dijk, Melanie M. van der Klauw, Anneke C. Muller-Kobold, Wilfred F.A. den Dunnen, Andre P. van Beek, Mark R. Postma

**Affiliations:** a Department of Neurosurgery, University of Groningen, University Medical Center Groningen, Groningen, The Netherlands; b Department of Radiology, Medical Imaging Center, University of Groningen, University Medical Center Groningen, Groningen, The Netherlands; c Department of Endocrinology, University of Groningen, University Medical Center Groningen, Groningen, The Netherlands; d Department of Pathology and Medical Biology, University of Groningen, University Medical Center Groningen, Groningen, The Netherlands; e Department of Laboratory Medicine, University of Groningen, University Medical Center Groningen, Groningen, The Netherlands.

**Keywords:** acromegaly, remission, transsphenoidal surgery

## Abstract

Transsphenoidal surgery (TSS) is the primary treatment of choice in acromegaly. Previous research on predicting remission after TSS was mainly based on clinical and radiological data, lacking histopathological evaluation. Our aim is to produce a clinicopathological prediction model for remission after TSS in acromegaly, enabling clinicians to better inform patients on expected treatment outcomes. This single-center study involved retrospective data collection in a large tertiary referral center in The Netherlands. One hundred patients in whom TSS was performed as primary therapy between January 2000 and July 2019 were included. Uni- and multivariate regression models were used to identify predictors of *early biochemical remission* according to the consensus criteria, *long-term* remission (defined as an age- and sex-normalized Insuline growth like factor 1and the absence of postoperative treatment (radiation therapy, repeat surgery, or medical therapy) at last follow-up, irrespective of residual tumor on postoperative magnetic resonance imaging ), and long-term remission without residual tumor (defined by the same biochemical and treatment criteria with the additional absence of residual tumor on postoperative magnetic resonance imaging ). In univariate logistic regression, a larger tumor volume, a larger maximum tumor diameter, a higher modified Knosp classification grade, the presence of cavernous sinus invasion, and a higher random growth hormone at diagnosis were significantly associated with lower remission rates. Tumor subtype and proliferation markers were not. In multivariate logistic regression, a simple model with only maximum tumor diameter as an independent variable predicted best for early biochemical remission (OR 0.85, 95% CI 0.78–0.93, *P* < .001, sensitivity 78.9%, specificity 71.8%), long-term remission (OR 0.79, 95% CI 0.70–0.88, *P* < .001, sensitivity 79.5%, specificity 73.7%), and long-term remission without residual tumor (OR 0.75, 95% CI 0.66–0.86, *P* < .001, sensitivity 79.4%, specificity 78.0%). Maximum tumor diameter was the best predictor, while histopathological characteristics were not predictive for remission after TSS in acromegaly in this cohort.

## 1. Introduction

Acromegaly has traditionally been viewed as a monomorphous disorder caused by a benign pituitary tumor. However, nowadays distinct subtypes are identified based on histopathological features.

The 2017 and 2022 WHO classifications of pituitary tumors have been an important step forward in the understanding of pituitary tumors, recognizing the role of transcription factors in tumor differentiation according to cellular lineage(s) and regulation of pituitary hormone production. These classifications are expected to provide more information on prognosis by improved identification of tumor types associated with aggressive behavior and elevated recurrence risk.^[1,2]^ Additionally, a clinicopathological classification has been proposed by Trouillas et al with grades based on invasion and proliferation, similar to other neuroendocrine tumors.^[3]^ This classification has demonstrated its prognostic worth by strongly predicting the probability of postoperative remission and has been validated in several independent cohorts with a variety of pituitary tumors.^[4,5]^

Radiological data has also proven useful in predicting therapeutic response. Several studies have demonstrated that somatotroph tumors hyperintense on magnetic resonance imaging (MRI) T2sequence, correlating with the sparsely granulated subtype, are less common, larger, and more likely to invade the cavernous sinus, and have lower Insuline growth like factor 1 (IGF-1) levels at diagnosis and lower remission rates in response to surgery and SSA compared to T2-hypointense tumors.^[6–8]^

Transsphenoidal surgery (TSS) is the primary treatment of choice in acromegaly. The overall surgical remission rate according to the 2010 criteria is 54.8% (95% CI 44.4–65.2%), for macroadenomas 52.7% (95% CI 41–64.4%), for invasive adenomas 29% (95% CI 20.1–37.8%), and for noninvasive adenomas 68.8% (95% CI 60–77.6%).^[9]^ It is important to identify patients in whom surgical cure is not attainable at an early stage, both to inform patients on expected treatment outcome and to select those who are more likely to need additional therapy.^[10]^ We previously demonstrated that maximum tumor diameter and random growth hormone (GH) concentration at diagnosis are the best predictors for remission after TSS in acromegaly.^[11]^ However, our research lacked data on tumor volume, T2-weighted MRI signal, and histopathological determinants (tumor subtype and proliferation markers).

To address this shortcoming, we added these parameters to the data from Groningen, aiming to determine their impact on attaining remission after TSS in acromegaly.

## 2. Methods

### 2.1. Study population

Retrospective data collection was performed on patients who received TSS as primary treatment for acromegaly between January 2000 and July 2019 in a large tertiary referral center in The Netherlands. The diagnosis of acromegaly was based on biochemical criteria valid at the time of diagnosis, i.e., failure of GH suppression to <1 μg/L during an oral glucose tolerance test (OGTT) and an elevation of the age- and sex-corrected IGF-1 standard deviation score of > 2 (above the upper limit of normal).^[12,13]^ The diagnosis was confirmed by histopathological examination of tissue obtained during surgery; a somatotropinoma was found in all patients.

### 2.2. Data were collected on

- Baseline demographic characteristics: sex, age at diagnosis, and surgery.
- Biochemistry: IGF-1, GH nadir during OGTT, and random GH at diagnosis, at 12 weeks to 1 year postoperatively and at last visit.
- Radiology: tumor size at diagnosis based on maximum diameter in millimeters (both as a dichotomous variable in terms of micro < 10 mm vs. macro ≥ 10 mm and as a continuous variable), tumor volume in cm^3^, cavernous sinus invasion according to the modified Knosp classification,^[14]^ T2-weighted MRI signal.
- Histopathology: tumor subtype (WHO 2017 classification) and proliferation markers (Ki-67, p53 and mitotic index).
- Preoperative medication categorized as SSA, GHRA, and DA.
- Applied postoperative treatments categorized as repeated surgery, radiotherapy, and medical therapy (subcategorized as SSA, GHRA, and DA).

### 2.3. Radiological data

An experienced neuroradiologist (HEW), a member of the multidisciplinary team of the Pituitary Center, reassessed the MRI examinations of the pituitary fossa on a Sectra workstation. All pituitary adenomas were classified according to the modified Knosp classification.^[14]^ Tumor volume (cm^3^) was determined by delineating the tumor circumference in 3 planes and, using software from AquariusNET Viewer (TeraRecon), the number of voxels within the tumor was multiplied by the voxel size. Tumor dimension (mm) was calculated in 3 directions. The T2-weighted signal intensity of the tumor was compared to the normal pituitary gland and, if not present, to the cortical gray matter of the temporal lobe and described as hypointense, isointense, or hyperintense.^[6]^ The T2-weighted signal intensity was also evaluated quantitatively by region of interest analysis of the pituitary adenoma and the normal pituitary gland or gray matter of the temporal lobe, and the ratio between them was used.

The postoperative MRI examinations of the pituitary fossa were investigated for the presence of certain tumor remnants, remnant location, and remnant dimensions.

### 2.4. Histopathological data

The histological cohort for this research is part of a larger series that includes surgically removed pituitary tumors sent to our Pathology department between January 2000 and July 2019. All tumors were subtyped according to the 2017 WHO classification, and proliferation markers were determined. Proliferation markers were defined as high or low according to the Trouillas classification (high: mitotic count > 2/10 HPF, Ki-67 index > 3%, and P53 > 10 strongly positive nuclei/ 10 HPF).^[3]^

Tissue microarrays (TMAs) that included the whole cohort were used for immunohistochemical analysis. In this article, only the data of the somatotroph tumors of this series are presented.

To construct the TMA blocks, the most representative area on the H&E slide was marked. Then, a cassette was filled with melted paraffin (Shandon Precision cut paraffin, Thermo) (60°C). The selected fields of interest were punched from the donor blocks with a 0.6 mm core punch (MTA1, Beecher Instruments, Inc). The punched tissue was then transferred into the wells within the recipient TMA blocks. A map with identification codes was used to assign the cores to the corresponding cases. The paraffin block with filled wells was then left for cooling at 4°C for 30 minutes. Sections with a thickness of 3 μm were cut from the TMA blocks. The TMA slides were then used for staining with Ki-67 (R.T.U. Ki-67, 30–9; Ventana) and P53 (BP53–11 Ventana RTU + optiview kit). The TMA slides were digitalized, with Philips IntelliSite Pathology Solution version 3.2 as the digital viewer program. Cores containing <50% tissue were excluded from evaluation. The Ki-67 percentages were generated using an image recognition program of virtual dual staining (see below). The p53 was scored as positive when 10 or more positive cells per high power field were counted. Staining for GH(1:2000 GH, rGH/1450; NSJ Bioreagents) and prolactin (1:800 PRL, PRL02; LSBio) was already performed and evaluated at the time of the original diagnosis for routine clinical purposes on whole sections.

Glass slides were scanned in a Philips Ultra-Fast Scanner 1.6 (Philips, Eindhoven, the Netherlands) with a 940-magnification lens, using a single-focus layer without Z-stacking. Tissue detection with focus points was applied automatically to obtain the optimal image. The platform used was Visiopharm Integrator System (VIS) version 2023.01.2.13695 (Visiopharm, Hørsholm, Denmark).

A training set (Tissue MicroArray TMA) of pituitary adenoma cases, which were identically handled and stained but not included in the current study, was used to calibrate tissue classification by Synaptophysin in Virtual Dual Staining (VDS) and nuclear classification of Ki-67. Calibration was performed in close collaboration with both platform vendors, independently of each other. In both platforms, VDS was applied to digitally align corresponding Ki–67 and Synaptophysin-stained sections. During this process, the algorithms automatically perform distortion and rotation modifications to eliminate small differences due to tissue and section processing. Alignment was verified visually for each case, and misaligned cases were excluded from further analysis. The algorithms were then set to use the Synaptophysin-stained area as the tumor classifier on the Ki–67 stained section. Within the whole core section, the complete tumor area was annotated. Tissue or staining artifacts were excluded. Ki–67 positivity was analyzed with nuclear classification algorithms which detect nuclei by morphological form and size and classify these as positive or negative based on pixel color and intensity. Ki–67 proliferation index was calculated by dividing the number of Ki–67 positive cells by the total number of positive and negative cells within the area classified as tumor by VDS.

A training set (TMA) of pituitary adenoma cases, which were identically handled and stained but not included in the current study, was used to calibrate tissue classification by 2 apps (Tissue detection and Tumor detection) and nuclear classification of Ki–67 also 2 apps (preprocessing and nuclear classification). The apps are calibrated and validated by Visiopharm.

### 2.5. Remission parameters

In the analysis of postoperative IGF-1 levels, only those values measured at least 12 weeks after surgery were analyzed. If these values were not available, patients were excluded from analysis. In patients who received SSA preoperatively, IGF-1 values were used that were measured at least 3 months after withdrawal of the medication, consistent with clinical guidelines regarding the reliability of IGF–1 measurements.^[12,15]^

### 2.6. Definition of remission

- Early biochemical remission defined based on the first available biochemistry 12 weeks to one year postoperatively according to the latest Consensus Statement on acromegaly therapeutic outcomes.^[15]^
  ○ An age- and sex-normalized IGF-1.
  ○ A random GH < 1 ug/L or nadir GH after oral glucose tolerance test (OGTT) < 0,4 ug/L.
  ○ Absence of postoperative treatment (radiation therapy, repeat surgery, or medical therapy) at this time point.
- Long-term remission, defined as an age- and sex-normalized IGF-1 at last visit and the absence of postoperative treatment (radiation therapy, repeat surgery, or medical therapy) until last follow-up. Due to lack of data on random GH and nadir GH during OGTT at last follow-up, it was decided not to use these parameters to define long-term remission.

### 2.7. Laboratory assays

IGF-1 concentrations were measured with a radioimmunoassay (RIA) of the Nichols Institute of Diagnostics, San-Juan Capistrano, CA, USA before 2002, the Nichols Advantage assay, standardized against the World Health Organization (WHO) International Reference Reagent for IGF-1 87/51, from 2002 to February 2006, the Immulite 2500 (Siemens) assay from 2006 to November 2011 and the Immulite 2000 (Siemens) assay from 2011 to April 2013. Since April 2013, IGF-1 concentrations were measured with the immunometric IDS-iSYS assay (Boldon, UK), the latter three assays standardized against the WHO International Standard for IGF-1, code 02/254.

For each IGF-1 assay, specific reference values were used.^[16]^ For the purpose of uniform reporting, IGF-1 results are expressed as age- and sex-corrected standard deviation scores (SDS).

GH concentrations were initially measured with the AutoDelfia immunoassay of Perkin Elmer LifeSciences (Turku, Finland), standardized against the WHO International Standard for Somatropin (80/505). Since January 2014, GH concentrations were measured with the immunometric IDS-iSYS assay (Boldon, UK), standardized against the WHO International Standard for Somatropin (98/574). GH concentrations (μg/L) were measured as a single random sample and expressed as an absolute value.

### 2.8. Statistical analysis

In descriptive statistics, differences were assessed with Fisher’s exact test for categorical variables and unpaired t-tests for continuous variables. If continuous variables were not normally distributed, a Mann–Whitney *U* test was performed. Data are expressed as mean ± SD, median (interquartile range), or percentages when appropriate. Two-tailed *P*-values < .05 were considered significant. Uni- and multivariate logistic regression coefficients and odds ratios were calculated to determine the relationship between both continuous and discrete independent (predictor) variables and each of the three predefined remission outcomes: early biochemical remission, long-term remission, and long-term remission without residual tumor. Sensitivity and specificity were calculated using the predicted probability of remission, with a probability threshold of 0.50 used to classify patients as having or not having remission; the area under the receiver operating characteristic curve (AUC) was calculated for each remission endpoint. For regression analyses, patients with missing values for any variable included in the respective model were excluded from that analysis (complete-case analysis). Missing data mainly concerned follow-up biochemistry and specific radiological or histopathological variables. Given the retrospective design and limited extent of missing data, complete-case analysis was considered appropriate; as the missingness mechanism could not be established, no imputation was performed. IBM SPSS Statistics 23 was used for data analysis.

## 3. Ethics statement

The study was conducted in accordance with the Declaration of Helsinki and approved by the Medical Ethics Review Board of the UMCG (protocol code 201900016 date of approval 25–03–2019).

INFORMED CONSENT STATEMENT: The study fulfilled all requirements for patient anonymity and was in agreement with regulations of our University Hospital for publication of patient data, as well as with the Dutch Civil Code (Article 458 on use of data for scientific research). Because the retrospective dataset dates back as far as 2000, requesting consent cannot reasonably be required. Requesting and obtaining consent would be more successful for more recent treatment periods but would lead to selection bias. For these reasons, the requirement for informed consent was waived by the Medical Ethics Review Board of the UMCG. Authors had access to information that could identify individual participants during or after data collection.

## 4. Results

### 4.1. Study population

In total, 123 patients were eligible for inclusion. Twenty-one patients were excluded due to missing biochemistry at diagnosis; 2 patients were excluded due to missing biochemistry as a result of insufficient follow-up between 12 weeks and 1 year after TSS. Subsequently, a retrospective cohort of 100 patients was available for analysis (supplementary figure 1, Supplemental Digital Content 1. Characteristics of the study population are shown in Tables 1 and 2. The median age at diagnosis was 51.9 years. Most patients (72%) presented with a macroadenoma with a median maximum tumor diameter of 15.4 mm and a median tumor volume of 4.0 cm^3^. Cavernous sinus invasion was present in 14%, and SSAs were prescribed preoperatively in 74% of patients. Median IGF-1 at diagnosis was 102.4 nmol/L, with a median relative reduction of 62.2% after surgery. Median follow-up was 11.8 years from diagnosis and 11.1 years from surgery. Early biochemical remission was achieved in 47% and long-term remission in 43% of patients. Applied postoperative treatment and presence of hypopituitarism at last visit are shown in supplementary table 1, Supplemental Digital Content 2.

**Table 1:** Patient characteristics.

Variable	Total (N = 100)
Sex	
Female	57 (57%)
Male	43 (43%)
Age	
At diagnosis (y)	51.9 (40.0; 61.1)
At surgery (y)	52.4 (40.7; 61.8)
Follow-up from diagnosis (yrs)	11.8 (6.3; 16.9)
Follow-up from surgery (yrs)	11.1 (6.1; 16.1)
Tumor size	
Macroadenoma	72 (72%)
Maximum tumor diameter (mm)–*23 missing*	15.4 (10.6; 21)
Tumor volume (cm^3^)–*25 missing*	4.0 (1.5; 6.4)
Cavernous sinus invasion (Knosp 3 or 4)–*23 missing*	14/77 (18%)
Trouillas grade *46 missing*	
1a	44/54 (81%)
1b	1/54 (2%)
2a	8/54 (15%)
2b	1/54 (2%)
Pre-operative medical treatment	76 (76%)
DA	11 (11%)
SSA	74 (74%)
GHRA	1 (1%)

Data are given as absolute numbers (percentage) or as median (interquartile range).

DA = dopamine agonist, SSA = somatostatin analog, GHRA = growth hormone receptor antagonist.

**Table 2:** Biochemical, radiological, and histopathological parameters.

Variable	
IGF-1	
At diagnosis (nmol/L)–*2 missing*	102.4 (80.3; 129.1)
Postoperatively (nmol/L)*–2 missing*	36.6 (26; 60.9)
Relative IGF-1 reduction (%)*–4 missing*	62.2 (45.2; 73.8)
Nadir GH during OGTT	
At diagnosis (µg/L)*–37 missing*	16.8 (5.7; 35)
Postoperatively (µg/L)*–46 missing*	0.4 (0.1; 1.4)
Relative nadir GH reduction (%)*–68 missing*	96.3 (77.8; 99.2)
Random GH	
At diagnosis (µg/L)*–15 missing*	23.4 (9.5; 50.1)
Postoperatively (µg/L)*–7 missing*	1.7 (0.5; 5.2)
Relative random GH reduction (%)–*20 missing*	89.9 (76.9; 97.5)
Early biochemical remission (2010 criteria)*–1 missing*	46/99 (47%)
Long-term remission *4 missing*	41/96 (43%)
MRI T2-signal intensity *30 missing*	
Hypointense	43/70 (61%)
Isointense	16/70 (23%)
Hyperintense	11/70 (16%)
Somatotroph subtype*—7 missing*	
Densely granulated	49/93 (53%)
Sparsely granulated	22/93 (24%)
Mammasomatotroph	20/93 (22%)
Mixed somatotroph-lactotroph	2/93 (2%)

Data are given as absolute numbers (percentage) or as median (interquartile range).

GH = growth hormone, IGF-1 = insulin-like growth factor 1.

### 4.2. Univariate analyses for selection of predictors for remission

Variables considered as predictors were selected based on the multicenter study from Coopmans and Postma et al^[11]^ All univariate analyses of possible predictors for remission are shown in Table 3. Higher GH at diagnosis (odds ratio [OR] 0.980, *P* = .008), larger tumor size (OR 0.196, *P* = .001), larger tumor volume (OR 0.857, *P* = .023), larger maximum tumor diameter (OR 0.849, *P* < .001), higher modified Knosp classification grade (OR 0.436, *P* < .001), and presence of cavernous sinus invasion (OR 0.054, *P* = .006) were significantly associated with lower remission rates. Possible histopathological predictors (subtype, mitotic figures, and Ki67 proliferation index) were not.

**Table 3:** Univariate analyses for selection of predictors for early biochemical remission.

Biochemistry	B	SE	*P*	% correct	OR
GH at diagnosis (µg/L)	−0,020	0,008	.008	70,6	0,980
IGF-1 at diagnosis (nmol/L)	−0,008	0,006	.179	59,8	0,992
Nadir GH during OGTT (µg/L)	−0,011	0,009	.254	58,1	0,989
Preoperative medication	−0,861	0,482	.074	59,6	0,423
Imaging					
Tumor size (micro or macro)	−1630	0,497	.001	66,7	0,196
Tumor volume (cm3)	−0,154	0,068	.023	71,6	0,857
Maximum tumor diameter (mm)	−0,164	0,046	<.001	75,3	0,849
Modified Knosp classification grade	−0,829	0,247	<.001	67,5	0,436
Cavernous sinus invasion	−2918	1069	.006	64,9	0,054
T2-weighted MRI signal	−0,157	0,320	.623	58,6	0,855
Histopathology					
Somatotroph subtype	0,081	0,239	.735	54,3	1084
Mitotic figures	−0,064	0,385	.868	54,0	0,938
Ki-67 proliferation index	−0,034	0,136	.799	55,7	0,966

GH = growth hormone, IGF-1 = insulin-like growth factor 1, OGTT = oral glucose tolerance test, OR = odds ratio, SE = standard error.

### 4.3. Multivariate analyses

Predictors that were significantly associated with lower remission rates in univariate analyses were selected for multivariate analyses (Table 4). In multivariate logistic regression, a simple model with only maximum tumor diameter as an independent variable best predicted both early biochemical remission (OR 0.849, 95% CI 0.776–0.928, *P* < .001, sensitivity 78.9%, specificity 71.8%), long-term remission (OR 0.787, 95% CI 0.702–0.882, *P* < .001, sensitivity 79.5%, specificity 73.7%), and long-term remission without residual tumor (OR 0.751, 95% CI 0.656–0.859, *P* < .001, sensitivity 79.4%, specificity 78.0%). Figure 1 shows the predicted probability of early biochemical remission by maximum tumor diameter. In this model, a tumor with a maximum diameter of 16 mm has a predicted 50% chance of early biochemical remission.

**Table 4:** Multivariate analyses (maximum tumor diameter in mm as independent variable).

Outcomes	% correct	Sensitivity	Specificity	OR	*P*	AUC
Early biochemical remission	75.3	78.9	71.8	0.849 (0.776–0.928)	<.001	0.790
Long-term remission	76.6	79.5	73.7	0,787 (0.702–0.882)	<.001	0.846
Long-term remission without residual tumor	78.7	79.4	78.0	0,751 (0.656–0.859)	<.001	0.862

AUC = area under the curve, OR = odds ratio.

**Figure 1.**
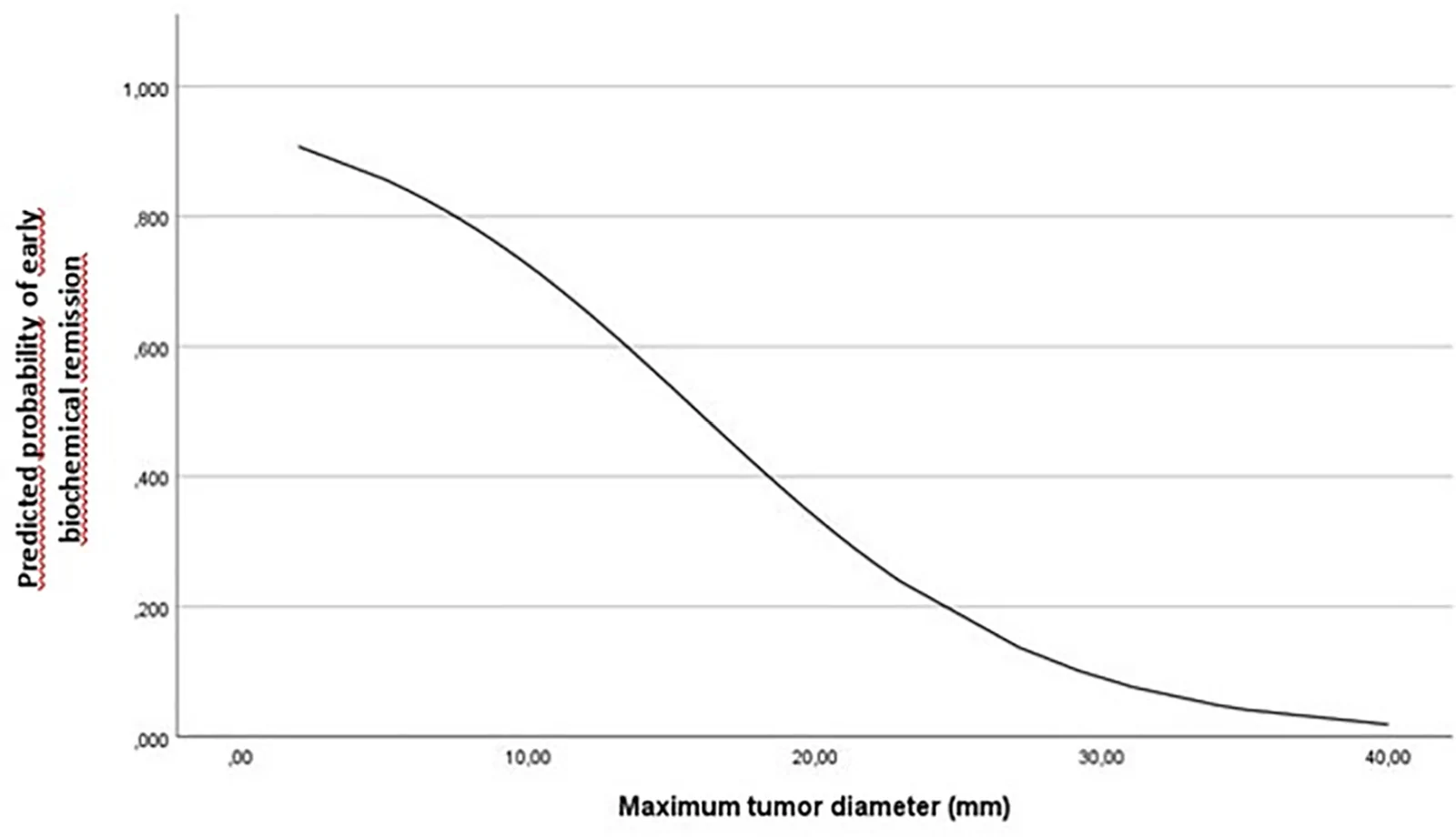
Predicted probability of early biochemical remission by maximum tumor diameter.

## 5. Discussion

This study’s main findings are that maximum tumor diameter at diagnosis is the best predictor of early biochemical remission, long-term remission, irrespective of residual tumor on postoperative MRI, and long-term remission without residual tumor after TSS for acromegaly, while histopathological characteristics were not predictive in this single-center cohort.

We previously demonstrated, in a model based on clinical data, that maximum tumor diameter and random GH concentration at diagnosis were the best predictors for remission after TSS in acromegaly.^[11]^ In this study, with added data on radiological (tumor volume, T2-weighted MRI signal) and histopathological determinants (tumor subtype, and proliferation markers i.e., Ki–67, p53 and mitotic index) to the data from Groningen. Preoperative prolactin levels were not systematically collected in this retrospective cohort and were therefore unavailable for consistent evaluation as a potential predictor of postoperative remission.

We found that histopathological characteristics were not predictive for surgical remission. This appears to be in contrast to the clinicopathological classification proposed by Trouillas et al^[3]^ and the subsequent validation studies,^[4,5]^ which show that combining clinical and histopathological data is useful for predicting disease- or recurrence/progression-free status. This discrepancy can probably be explained by the comparatively low number of tumors with positive proliferation markers in our study (n = 1 for mitotic count > 2/10 HPF, n = 3 for Ki–67 index > 3%, and n = 1 for P53 > 10 strongly positive nuclei/10 HPF, data not shown). There were only 2 cases that were classified as proliferative according to the Trouillas classification, which is substantially lower than in the Lyon cohort, performed in a center of reference for aggressive pituitary tumors and carcinomas. The results therefore indicate an absence of demonstrable incremental predictive value in this dataset, rather than evidence that histopathological characteristics have no prognostic relevance. Our results further support the robustness of maximum tumor diameter as a readily available preoperative predictor, while indicating that additional histopathological characteristics may require larger cohorts to determine their potential prognostic value.

Interestingly, this study confirms the importance of radiological findings. However, cavernous sinus invasion, classified using the modified Knosp classification, was not found to be a stronger predictor for remission in our multivariate model than maximum tumor diameter in any plane. It is well known that lateral extension is generally the main factor determining whether gross total resection of a pituitary tumor is feasible. Here, the statistical phenomenon of imperfect multicollinearity comes into play: cavernous sinus invasion and maximum tumor diameter have a nearly exact linear relationship. However, the continuous variable of maximum tumor diameter (in mm) has a much higher information density than the categorical variable of cavernous sinus invasion (present vs absent). As maximum tumor diameter increases, the chance of cavernous sinus invasion (Knosp 3 or 4) increases as well, making the latter variable statistically redundant. Our results indicate that once maximum tumor diameter is known, the model correctly predicts long-term remission without residual tumor in 78.7% of cases. Knosp grading does not increase that percentage any further.

A novel radiological metric, the MI Ratio, and its potential for predicting surgical outcome in acromegaly, has recently been described.^[17]^ This ratio is defined as the distance from the midline to the lateral maximum of a tumor, divided by the distance between the 2 cavernous carotid arteries on coronal MRI. It is a continuous variable that provides information on lateral extension in mm, and it may therefore be a stronger predictor for remission than both maximum tumor diameter and Knosp grade.

Recent studies also have shown that in functioning pituitary tumors, there is a higher rate of invasion of the MWCS, which is not accurately reflected by the modified Knosp classification grade.^[18–20]^ Mohyeldin et al described tumor invasion of the medial wall of the CS in somatotroph adenomas in 25% in Knosp grade 0, 76% in grade 1 and 100% in grades 2 to 4.^[18]^ It is important to note that invasion of the medial wall of the CS can therefore have an attenuating effect on the relationship between Knosp grade and remission rates.^[20]^ Additional resection of the medial wall of the CS may result in higher remission rates, a topic currently under investigation in our center.

The strengths of this study are: the availability of an extensive dataset, all MRIs were reassessed by an experienced neuroradiologist, all histopathological data were assessed by an seasoned neuropathologist using artificial intelligence software to generate Ki67 percentages, and all patients were operated on in an experienced tertiary surgical center by just three experienced neurosurgeons. This is relevant as it is well known that clinical outcomes of TSS are related to surgical experience and practice volume.^[21]^

This study’s main limitations are its limited sample size and its single-center, retrospective design. The assays measuring GH and IGF-1 have changed several times over the years. This might influence the outcome. Although the findings of this study are largely consistent with a previous multicenter analysis we performed,^[11]^ it is most probably due to the smaller sample size that random GH concentration at diagnosis was not found to be associated with a lower chance of long-term remission in multivariate analysis. All procedures were performed at a tertiary referral center by 3 experienced neurosurgeons, which may limit the generalizability of our findings to centers with different levels of surgical experience, because it is well known that surgical experience influences the outcomes of TSS.

In conclusion, maximum tumor diameter was found to be the best predictor in this single-center cohort, while histopathological characteristics were not significantly associated with remission in this cohort.

## Author contributions

**Data curation:** Rob A. Vergeer, Henriette E. Westerlaan, Annet Willems, Anke D. van den Beukel, Friederike E. Roelandt-Schumann, Henk J. Buikema, Anneke C. Muller-Kobold, Wilfred F.A. den Dunnen.

**Writing – original draft:** Rob A. Vergeer, Mark R. Postma.

**Formal analysis:** Anke D. van den Beukel, Mark R. Postma.

**Writing – review & editing:** Friederike E. Roelandt-Schumann, Jos M.A. Kuijlen, J. Marc C. van Dijk, Melanie M. van der Klauw, Anneke C. Muller-Kobold, Wilfred F.A. den Dunnen, Andre P. van Beek.

**Conceptualization:** Wilfred F.A. den Dunnen, Andre P. van Beek, Mark R. Postma.

**Supervision:** Andre P. van Beek.




