# Supplementary material for: A clinicopathological prediction model for remission after transsphenoidal surgery for acromegaly: A retrospective single-center cohort study
Source: Medicine (Baltimore). 2026 Nov 6;105(45):e51358. doi: 10.1097/MD.0000000000051358 (PMC13747415; doi:10.1097/MD.0000000000051358)
Supplement: Supplementary file 1 [file medi-105-e51358-s002.docx]

**Supplementary Table 1. Applied postoperative treatment and presence of hypopituitarism at last visit (data are given in percentages.**

| Total number of patients | 100 |
| --- | --- |
| **Postoperative treatment** |  |
| Any | 48 |
| Second surgery | 18 (16 TSS, 2 craniotomy) |
| Third surgery | 2 (all TSS) |
| Radiotherapy | 29 |
| Medical therapy  DA  SSA1  SSA2  GHRH | 47  14  45  6  23 |
| **Medical therapy at last visit** |  |
| Any  DA  SSA1  SSA2  GHRH | 34  5  24  4  15 |
| **Hypopituitarism at last visit** |  |
| Any Axis  Thyroid axis  Adrenal axis  Gonadal axis  Somatotrophic axis  Central diabetes insipidus | 34  16  17  15  0  4 |

TSS, transsphenoidal surgery; DA, dopamine agonist; SSA1, first generation somatostatin analog; SSA2, second generation somatostatin analog; GHRA, growth hormone receptor antagonist;
